# Pyroptosis in spinal cord injury

**DOI:** 10.3389/fncel.2022.949939

**Published:** 2022-11-17

**Authors:** Jian Yin, Ge Gong, Wenhui Wan, Xinhui Liu

**Affiliations:** ^1^Department of Orthopedics, The Affiliated Jiangning Hospital With Nanjing Medical University, Nanjing, China; ^2^Department of Orthopedics, Shanghai General Hospital of Nanjing Medical University, Shanghai, China; ^3^Department of Geriatrics, Jinling Hospital, Medical School of Nanjing University, Nanjing, China

**Keywords:** spinal cord injury, pyroptosis, inflammasome, gasdermins, regulated cell death

## Abstract

Spinal cord injury (SCI) often brings devastating consequences to patients and their families. Pathophysiologically, the primary insult causes irreversible damage to neurons and glial cells and initiates the secondary damage cascade, further leading to inflammation, ischemia, and cells death. In SCI, the release of various inflammatory mediators aggravates nerve injury. Pyroptosis is a new pro-inflammatory pattern of regulated cell death (RCD), mainly mediated by caspase-1 or caspase-11/4/5. Gasdermins family are pore-forming proteins known as the executor of pyroptosis and the gasdermin D (GSDMD) is best characterized. Pyroptosis occurs in multiple central nervous system (CNS) cell types, especially plays a vital role in the development of SCI. We review here the evidence for pyroptosis in SCI, and focus on the pyroptosis of different cells and the crosstalk between them. In addition, we discuss the interaction between pyroptosis and other forms of RCD in SCI. We also summarize the therapeutic strategies for pyroptosis inhibition, so as to provide novel ideas for improving outcomes following SCI.

## Introduction

Spinal cord injury (SCI) is a devastating injury, which results in serious motor and neurological dysfunctions and places tremendous financial burdens on patients. Statistics show that the costs for patients with SCI are staggering at $14.5 billion in American (Habtemariam, 2019) and $2.67 billion in Canada per year (Katoh et al., 2019). Despite clinical urgent need, no effective treatment to date is uniquely specific for SCI. Thus, further understanding of the potential mechanisms of SCI, especially the pattern of cell death, is needed to explore underlying therapeutic targets.

The pathophysiology of SCI involves two stages: (i) the primary injury, due to mechanical injury to spinal cord and leading to destruction of neural parenchyma, including cell membrane rupture and tissue necrosis (Mortezaee et al., 2018), and (ii) the secondary injury, triggering by the primary injury and producing deleterious contents, involving glutamate, potassium, K^+^ flux, reactive oxygen species (ROS), and cathepsin B (Dixon, 2017). These contents are highly pro-inflammatory, and initiate a persistent secondary injury cascade. This cascade leads to inflammatory reaction of neurons and glial cells, ER, mitochondrial dysfunction and excitotoxicity, and ultimately changes in organization and structural architecture of spinal cord and permanent neurological deficits as a result of axonal destruction, demyelination and neuronal death (Alizadeh et al., 2019). Accordingly, inflammation is the key regulator in SCI.

Cell death plays a vital role in the pathophysiology of SCI. Three different patterns of cell death in SCI, including apoptosis, autophagy death, and necrosis have been extensively studied in the past (Tang et al., 2014; Bao et al., 2019). Recently, researchers have defined a new type of regulated cell death (RCD): pyroptosis, a pro-inflammatory (infectious and sterile) form of lytic cell death, which promotes cell swelling, plasma membrane dissolution, chromatin fragmentation, and release of inflammatory cytokines, such as interleukin (IL) –18 and IL-1β, through gasdermins pores. The pathophysiology of SCI involves two stages: (i) gasdermins plasma membrane pore formation and releasing of IL-1β/18 and ionic fluxes, and (ii) subsequent plasma membrane rupture (PMR), cell swelling and lysis, and release of LDH (Kayagaki et al., 2021). Neuroinflammation, a double-edged sword in SCI, involves numerous cell types such as microglia, astrocytes, neurons, and pericytes (Miron and Franklin, 2014). After SCI, these cells and the multicellular interactions are interrupted and disorganized, resulting in impaired neurological recovery. Substantial evidence indicates inflammation is an important determinant in the pathogenesis of SCI, particularly, pyroptosis has emerged as a critical factor. Thus, understanding the mechanism of pyroptosis on SCI provides new therapeutic approaches for clinical treatments. This review focuses on recent findings on the mechanisms and function of pyroptosis in SCI, especially the interactions between different cell lines as well as the crosstalk between different RCD patterns. This article also highlights the potential drugs that can alleviate pyroptosis, so as to provide new ideas for the potential treatment of SCI.

## Overview: Pyroptosis

Friedlander (1986) found that anthrax lethal toxin could cause the death of macrophages and the rapid release of intracellular contents, which may be the earliest description of pyroptosis. In Zychlinsky et al. (1992) found cell death in macrophages infected with Shigella flexneri, which was different from apoptosis. The term pyroptosis was initially coined by Cookson and Brennan (2001) in to describe a form of macrophage death suffering Salmonella infection. Pyroptosis is a lytic, pro−inflammatory RCD program and characterized by its reliance on gasdermin proteins as the executioners of cell death. Activation of pyroptosis has been classified into a canonical pathway and non-canonical pathway. Generally, in a canonical pyroptotic pathway, activation of inflammasome occurs through a multitude of pathogen-associated molecular patterns (PAMPs) or damage-associated molecular patterns (DAMPs), including nucleic acids, flagellin, bacterial toxins, microbial cell wall components, alum, ATP, and uric acid. Inflammasome assembly is triggered when sensor proteins recognize their ligands. This nucleates the inflammasome and initiates polymerization of the scaffolding protein apoptosis-associated speck-like protein containing a CARD (ASC), a non-enzymatic scaffolding protein couples the sensor protein to the effector protease. Cross-linked ASC fibers recruit pro-inflammatory caspases that undergo proximal-induced autoactivation for proteolytic activity (Franklin et al., 2018). Upon activation, inflammasomes recruit caspase-1 via CARD-CARD interactions. The caspase-1 is localized on inflammasomes and clustering of caspase-1 generates a transient caspase-1 p33/p10 species, obligated to most substrate cleavage events. Cleavage of the CARD domain from the p33 fragment generates a caspase-1 p20/p10 species that speedily detaches from the inflammasome and becomes inactive (Boucher et al., 2018).

Inflammasomes, large cytosolic multiprotein complexes that detect a broad range of PAMPs and DAMPs and serve as platforms upon which activated proinflammatory caspases cleave and activate their protein substrates (Broz and Dixit, 2016). Depending on the type of caspases involved, the inflammasome are also divided into typical and atypical inflammasomes (Zhou C. et al., 2022). Canonical inflammasome complex is composed of three components, including a sensor protein, a caspase-1 family protease and an ASC. Danger signals such as extracellular ATP and mitochondrial dysfunction are potent inflammasome activators during SCI (Rathinam and Fitzgerald, 2016). Assembly of the inflammasome complex leads to caspase-1 activation by an autoproteolysis process. In addition to the cleavage of precursor cytokines pro-IL-1β/18 into IL-1β/18, the activated caspase-1 substrates also involve in the cleavage of Gasdermin D (GSDMD) into N-terminal fragment of GSDMD (GSDMD-NT), which inserts pores in the cell membrane and compromises membrane integrity, accompanied by the secretion of inflammatory signaling molecule, including IL-1β/18 (Liu et al., 2016). Additionally, non-canonical inflammasome directly senses cytosolic LPS from Gram-negative bacteria and their outer membrane vesicles through caspase-4/5 in humans or caspase-11 in mice. Like caspase-1, activated caspase-4/5/11 cleave GSDMD to induce pyroptosis (Shi et al., 2015). Notably, while caspase4/5/11 cannot process pro-IL-1β/18 directly, they can facilitate the maturation and secretion of the IL-1β/18 via the activation of the NLRP3 in an indirect way (Humphries et al., 2018; Yang et al., 2022). The receptors in the inflammasome complex belong in most cases to the Nod-like receptor (NLR) and absent in melanoma (AIM) 2-like receptor (ALR) families. Currently, known inflammasomes include the NLRP1, NLRP2, NLRP3, NAIP/NLRC4, NLRC5, NLRP6, NLRP12, AIM2, and pyrin (Linder et al., 2020). The best-studied inflammasomes in central nervous system (CNS) are NLRP1, NLRP3, and AIM2, although NLRC4 is also identified (Zendedel et al., 2018; Han et al., 2019). NLRP1, the first inflammasome identified, remains somewhat mysterious owing to its unique activation mechanism (Lamkanfi and Dixit, 2014). NLRP1 can be activated by intracellular ATP depletion, ion flux downstream of the P2 × 4/7 receptors and extracellular Aβ (Chavarría-Smith et al., 2016). Whereas NLRP1 in neurons is different from macrophages because it contains inhibitors of caspase 11 and apoptosis protein X-linked inhibitor of apoptosis protein (XIAP) (Mortezaee et al., 2018). The activation mechanism of caspase-11 in CNS injury remains unclear. Whether activation of the NLRP1 inflammasome induces cleavage of caspase-11 or caspase-11 forms independent of caspase-1 are still under investigation. Among SCI studies, NLRP3 inflammasome is the most extensively studied, which is expressed in microglia, neuron and astrocytes and detects perturbations to cellular homeostasis, although the unifying mechanism for NLRP3 activation remains controversial (Lamkanfi and Dixit, 2014). NLRP3 can be selectively blocked by concentrated extracellular K^+^, MCC950, ketogenic diet, glyburide, and minocycline (Muñoz-Planillo et al., 2013; Wallisch et al., 2017). AIM2 is a non-NLR inflammasome, which recognizes double-stranded DNA (dsDNA), either pathogen or host-derived, through its C terminal HIN200 domain (Lamkanfi and Dixit, 2014). Widespread necrotic cell death after SCI releases cell-free DNA into the cerebrospinal fluid, which may act as a ligand for AIM2. Binding of dsDNA to the HIN domain of AIM2 recruits ASC and procaspase-1, leading to the maturation of caspase-1, and pro-inflammatory cytokines (de Zoete et al., 2014).

**FIGURE 1:**
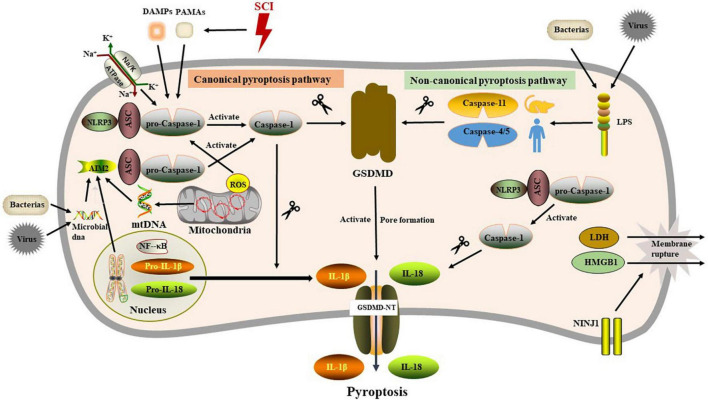
The molecular mechanism of pyroptosis.

Gasdermins (GSDMs) are a family of porogens containing six gene clusters [GSDMA-E and pejvakin (PJVK)] (Qin et al., 2022), and activated by proteolytic removal of autoinhibitory carboxy-terminal domains, typically by caspase modulators (LaRock et al., 2022). To date, little was known about GSDMs other than GSDMD. GSDMA is present in mammals, reptiles and birds. Notably, GSDMB, GSDMC, and GSDMD are only expressed in mammals (Broz et al., 2020), intriguingly, unlike other GSDMs, GSDMB lacks a mouse ortholog, which hinders the study of its *in vivo* function (Ivanov et al., 2022). Another unique feature of GSDMB is that it binds to phosphatidylinositol phosphate and sulfide (Chao et al., 2017). In epithelial cells, GSDMB can be cleaved by GzmA to induce pyroptosis (Zhou et al., 2020). GSDMD, one member of GSDMs family, is the major driver of pyroptosis, which contains dual domain with autoinhibitory mechanism, whereby the C terminal domain possessing repressor activity constitutively interacts with and represses the N terminal domain possessing pore-forming activity (Liu et al., 2018). The dual domain structure of GSDMD in humans is linked by a domain containing a caspase-1/4 (or caspase-1/11 in mice) cleavage site (Shi et al., 2015; Kovacs and Miao, 2017). Upon cleavage by caspase-1/4 (or caspase-1/11 in mice), the autoinhibition of the C terminus is eliminated, releasing the p30 N terminal fragment with pore-forming activity (GSDMD-N). Lipid binding triggers the oligomerization of GSDMD-N to form transmembrane pores in the plasma membrane, leading to disruption flocal osmotic potential, swelling, and terminal membrane rupture (Liu et al., 2016). GSDMD pore (10–15 nm in diameter) provides a logical conduit through which IL-1β (4.5 nm in diameter) and IL-18 (5.0 nm in diameter) escape the cell (Kovacs and Miao, 2017). Except for GSDMD, recent study implicates that chemotherapy drugs could induce pyroptosis through caspase-3 cleavage of GSDME, another member of the gasdermins family (Wang et al., 2017). The content described below, unless otherwise specified, refers to GSDMD-mediated pyroptosis (Figure 1).

Ninjurin (NINJ) is a cell adhesion molecule and membrane protein with homophilic binding properties. Studies have shown that its expression is increased in neurons and Schwann cells of distal nerve segments after nerve transection or crush injury and promotes axonal sprouting and nerve repair in rat sciatic nerves (Araki and Milbrandt, 1996; Araki et al., 1997). In mammals, NINJ family is divided into NINJ1 and NINJ2. It has been reported that NINJ1 is involved in inflammatory processes. After acute cerebral ischemia in rats, NINJ1 is dynamically expressed in a variety of immune cells and involved in cell trafficking and axonal growth following neuroinflammation (Lee et al., 2016, 2018). Similarly, expression level of NINJ1 was up-regulated in the injured spinal cord in a rat model of spinal cord crush injury. As touched upon earlier, PMR is a catastrophic event in the second stage of pyroptosis, a process distinct from GSDMs-dependent pore formation, which is closely related to the regulation of NINJ1 (Kayagaki et al., 2015; Rühl and Broz, 2022). NINJ1 releases LDH and intracellular DAMPs, such as HMGB1, by modulating protrusive membrane dynamics. NINJ1 silencing (NINJ1^–/–^) can attenuate PMR and its associated inflammatory dissemination response. Of note, NINJ1 is dispensable for the formation of GSDMs-dependent pore (Ahn et al., 2014; Kayagaki et al., 2021). Intriguingly, NINJ2, a homolog of NINJ1, is identified as a key pro-survival factor in neurons and glial cells, which can be targeted for silencing by microRNA-764 (miR-764) (Jing et al., 2018). Although NINJ has a central role in both the nervous system and pyroptosis, the mechanism of NINJ in SCI deserves further investigation.

**FIGURE 2:**
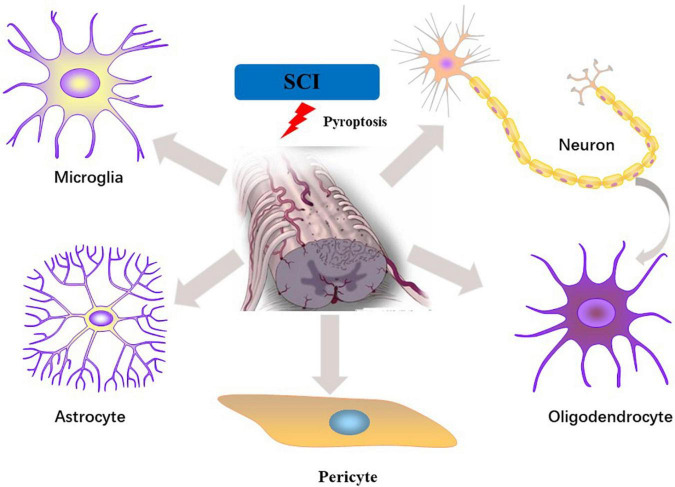
Pyroptosis in different cell types of the spinal cord following SCI.

IL-1β/18 are fully studied substrates of proinflammatory caspases. Release of IL-1β/18 from cells is a widely used assay for inflammasome induction. Despite closely related to pyroptosis, cleavage and release of IL-1β/18 is neither necessary nor sufficient for the occurrence of pyroptosis (McKenzie et al., 2018). In non-canonical pyroptotic pathway, mouse caspase-11 and human caspase-4/5 are sensitized by intracellular lipopolysaccharide (LPS) generated by bacteria, resulting in pyroptosis through the cleavage of GSDMD. In the process of pyroptosis, IL-1β and IL-18 are released into the extracellular environment, which elicit intensive inflammatory reactions via activating the IL-1R/IL-18R-MyD88-NFκB pathway. The other inflammatory stimulators, such as IL-1α and HMGB1, released during pyroptosis also act to magnify the inflammatory reactions. During SCI, inflammasome activation is initiated by acute perturbations in homeostasis (e.g., ion flux, mitochondrial dysfunction) together with danger signals in the microenvironment (e.g., extracellular ATP, double-stranded DNA, microbial molecules) (de Rivero Vaccari et al., 2016). Microglia, neuron and oligodendrocytes, etc. may undergo pyroptosis in response to stimuli. Here we specifically focus upon evidence for pyroptosis in each cell populations of spinal cord (Figure 2 and Table 1).

**TABLE 1:** SCI experiments centered on pyroptosis.

Therapeutic target/agents	Animal	Cell type	SCI model	*In vitro* cell model	Caspase	Gasdermins	Inflammasome	Main mechanisms	References
CD73	C57BL/6J mice	Microglia	Squeeze model at T8–T9 with a forceps to a depth of 0.2 mm for 20 s	BV2 microglia stimulated by LPS	Caspase-1	GSDMD	NLRP3	CD73 reduces GSDMD transcription through the adenosine A_2B_ adenosine receptor/PI3K/AKT/foxo1 pathway. In addition, CD73 and HIF-1α form a positive feedback regulatory loop.	Xu et al., 2021
TLR4/lncRNA-F630028O10Rik	C57BL/6 mice	Microglia	Squeeze model at T8–T9 with a forceps to a depth of 0.2 mm for 20 s	BV2 microglia stimulated by LPS	Caspase-1	GSDMD	NLRP3	Activated TLR4 after SCI promotes the expression of lncRNA-F630028O10Rik. LncRNA-F630028O10Rik/miR-1231-5p/Col1a1 enhances pyroptosis through the PI3K/AKT pathway.	Xu et al., 2020
TLR4 knockout	C57BL/6 mice	Microglia	Squeeze model at T8–T9 with a forceps to a depth of 0.2 mm for 60 s	BV2 microglia stimulated by LPS + ATP	Caspase-1	GSDMD	NLRP3	TLR4 promotes the expression of dead-box helicase 3 X-linked (DDX3X) by activating the JAK2/STAT1 signaling pathway, thereby promoting pyroptosis.	Wang et al., 2022
AOPPs/Apocynin	SD Rats	Microglia	Hemi-contusion injury model at C5 with a displacement of 2.2 mm	BV2 microglia stimulated by AOPPs/MAS or LPS	Caspase-1	GSDMD	NLRP3	AOPPs lead to overproduction of ROS, phosphorylation of p38 MAPK and JNK, and triggering the nuclear translocation of NF-κB p65 to induce pyroptosis by activating NADPH oxidase. Apocynin, an NADPH oxidase inhibitor, inhibits this effect of AOPPs.	Liu et al., 2020
Kaempferol	SD Rats	Microglia	Hemi-contusion injury model at C5 with a displacement of 2.0 mm	BV2 microglia stimulated by LPS and ATP	Caspase-1	GSDMD	NLRP3	Kaempferol reduces ROS production by inhibiting NADPH oxidase 4, phosphorylation of p38 MAPK and JNK along with nuclear translocation of NF-κB p65, ultimately inhibiting pyroptosis.	Liu et al., 2021
Nrf2	SD Rats	Microglia	Collision model at T10with by an impactor	BV2 microglia stimulated by LPS and ATP	Caspase-1	GSDMD	NLRP3	Nrf2 inhibits GSDMD transcription and pyroptosis by promoting the expression of miR-146a.	Zhang D. et al., 2022
Celastrol	SD Rats	Microglia	Squeeze model at T8–T10 with a 30 g forces vascular clip for 1 min	BV2 microglia stimulated by LPS and ATP	Caspase-1	GSDMD	NLRP3	Celastrol inhibits pyroptosis by inhibiting the expression of NF-κB/p-p65.	Dai et al., 2019
Polydatin	SD Rats	Microglia	Collision model at T8 with by a 10 g impactor falling from a height of 5 cm	BV2 microglia stimulated by LPS	Caspase-1	GSDMD	NLRP3	Polydatin attenuates NLRP3 activation by inhibiting iNOS expression.	Lv et al., 2019
Paeonol	SD Rats	Microglia	Squeeze model at T9 with a 30 g forces vascular clip for 1 min	BV2 microglia stimulated by LPS and ATP	Caspase-1	GSDMD	NLRP3	Paeonol inhibits pyroptosis accompanied by inhibition of TLR4, MyD88 and P-p65/p65 expression.	Zhao et al., 2022b
CORM-3	SD Rats	Neuron	Squeeze model at T9 with a 30 g forces vascular clip for 1 min	Neurons suffering OGD	Caspase-1	GSDMD	NLRP1 NLRP3	CORM-3 alleviates pyroptosis by inhibiting the activation of IRE1 and its downstream XBP1.	Zheng et al., 2019
MCC950	C57BL/6 mice	Neuron	Squeeze model at T6–T7 with a vascular clip for 1 min	Neurons suffering OGD or LPS	Caspase-1	*Not detected*	NLRP3	MCC950 attenuates neuroinflammation after SCI by blocking NLRP3 inflammasome assembly.	Jiao et al., 2020
Metformin	SD Rats	Neuron	Collision model at T9–T10 with by a 10 g impactor falling from a height of 2.5 cm	Neurons stimulated by LPS	Caspase-1	*Not detected*	NLRP3	Metformin inhibits pyroptosis by activating phosphorylated AMPK.	Yuan et al., 2022
Zinc	C57BL/6 mice	Neuron	Collision model at T9 with by a 12.5 g impactor falling from a height of 5 cm	VSC4.1 cells treated with hydrogen peroxide	Caspase-1	*Not detected*	NLRP3	Zinc inhibits oxidative damage and NLRP3, possibly by activating the Nrf2/HO-1 pathway.	Li D. et al., 2020
LiCl	SD Rats	Neuron	Collision model at T10 with by a 10 g impactor falling from a height of 2.5 cm	PC12 cells suffering OGD	Caspase-1	GSDMD	NLRP3	Lithium alleviates pyroptosis by upregulating Nrf2/HO-1 pathway.	Zhao et al., 2022d
AIM2 knockdown	C57BL/6 mice	Neuron	I/R model	Neurons stimulated by CSF from I/R mice	Caspase-1	*Not detected*	AIM2	Increased dsDNA release after SCI activates AIM2 inflammasome. AIM2 knockdown inhibits AIM2 activation.	Li et al., 2019
lncRNA H19 knockdown	C57BL/6 mice	Neuron	I/R model	PC12 cells suffering OGD	Caspase-1	*Not detected*	NLRP3	LncRNA H19 suppressed miR-181a-5p, thereby increasing HMGB1 and promoting pyroptosis. lncRNA H19 knockout reverses this effect.	Guo et al., 2022
BMSC-EXOs; circ_003564	SD Rats	Neuron	Collision model at T10 with by a 10 g impactor falling from a height of 12.5 mm	Neurons stimulated by H_2_O_2_	Caspase-1	GSDMD	NLRP3	BMSC-EXOs alleviate pyroptosis by delivering circ_003564.	Zhao et al., 2022c
Smad3 inhibitor	ICR mice	Neuron	Squeeze model at T10 with a vascular clip for 1 min	*Not established*	Caspase-1	*Not detected*	Denied AIM2 and NLPR1	Smad3 inhibitor can inhibit pyroptosis by downregulating caspase-1, which may be independent of AIM2 and NLPR1.	Zhu et al., 2020
Quercetin	C57BL/6 mice	Microglia and neuron	LPS-induced PD and depression mouse model	BV2 microglia stimulated by LPS and ATP	Caspase-1	GSDMD	NLRP3	Quercetin promotes microglia mitophagy, reduces mtROS accumulation and NLRP3 activation, and ultimately alleviates neuronal damage.	Han et al., 2021
Hv1 knockout (*in vivo*); N-acetyl cysteine (*in vitro*)	C57BL/6 mice	Microglia and PC12 cell	Collision model at T10 with by a 5 g impactor falling from a height of 11 mm	PC12 cells suffering OGD/R	Caspase-1	GSDMD	NLRP3	Hv1 deficiency reduces microglial ROS production, which in turn reduces neuronal NLRP3 activation and pyroptosis.	Li X. et al., 2020
17β-estradiol	SD Rats	ODCs and Microglia	Squeeze model at T8 with a 50 g plate for 5 min	*Not established*	Caspase-1	*Not detected*	NLRP3, NLRP1b	17β-estradiol alleviates microglial hyperplasia and oligodendrocyte injury by inhibiting NLRP3, NLRP1b, and caspase-1.	Zendedel et al., 2018
Non-use	SD Rats	OPCs	Collision model at T10 with impactor	*Not established*	Caspase-2	*Not detected*	NLRP3	The expression of NLRP3, ASC and caspase-2 in OPCs after SCI was significantly higher than that in astrocytes, which may be related to the high mortality of OPCs.	Yanagisawa et al., 2019
NLRP6 silence	SD Rats	Astrocytes and neuron	*Not established*	OGD/R	Caspase-1	GSDMD	NLRP6	NLRP6 is mainly expressed in astrocytes. NLRP6 activates caspase-1 to induce neuronal pyroptosis in primary neuron-astrocyte co-cultures.	Zhang et al., 2020
BMSC-EXOs	SD Rats	Pericyte	Collision model at T10 with impactor	Spinal cord microvascular pericyte stimulated by IFN-γ, TNF-α, LPS, Lipo, and ATP	Caspase-1	*Not detected*	Nod1	BMSC-EXOs protect pericytes by inhibiting the Nod1 inflammasome and pyroptosis, thereby improving the blood-spinal cord barrier and promoting neuronal survival.	Zhou Y. et al., 2022
Piperine	C57BL/6 mice	Neuron	Squeeze model at T8 with a vascular clip for 1 min	*Not established*	Caspase-1	GSDMD	NLRP3	Piperine promotes neurological recovery after SCI by activating autophagy as well as inhibiting oxidative stress and pyroptosis.	Zhang H. et al., 2022
Betulinic acid	C57BL/6 mice	Neuron	Collision model at T11–T12 with by a 15 g impactor falling from a height of 15 mm	*Not established*	Caspase-1	GSDMD	NLRP3	Betulinic acid promotes autophagy through the AMPK-mTOR-TFEB signaling pathway, thereby eliminating ROS accumulation and inhibiting neuronal pyroptosis.	Wu C. et al., 2021
Baicalein	C57BL/6 mice	Neuron	I/R model by blocking the aortic arch for 10 min	*Not established*	Caspase-1	GSDMD	NLRP3	Baicalein alleviates pyroptosis and endoplasmic reticulum stress-mediated apoptosis by activating autophagy.	Wu et al., 2020

## Pyroptosis of different cell types following spinal cord injury

### Microglia

Activated microglia are major innate immune cells in the spinal cord and play an important role in neuroinflammation (Walsh et al., 2014; David et al., 2015). Microglia are quickly activated after SCI, resulting in increased expression of pro-inflammatory cytokines mediated by multiple signaling cascades, while hyperactivation of microglia causes overproduction of pro-inflammatory cytokines and ROS, which in turn aggravates the inflammatory response and secondary injury (Gaudet and Fonken, 2018).

Activation of the inflammasome is a key step in neuroinflammation induced by secondary injury in SCI. CD73 is an immunosuppressive molecule that regulates the conversion of extracellular ATP to adenosine, maintaining immune homeostasis by regulating the balance between pro-inflammatory ATP and immunosuppressive adenosine to prevent excessive immune responses (Antonioli et al., 2013; Yu et al., 2018). Xu et al. (2021) observed that NLRP3 and GSDMD expression were significantly up-regulated in blood samples isolated from SCI patients. In a mouse spinal cord squeezed injury model, it was found that CD73 inhibits microglial pyroptosis by suppressing the expression of GSDMD at the transcriptional level in Foxo1 via the PI3K/AKT signaling axis. CD73 knockout exacerbated SCI, while CD73 overexpression can alleviate microglial pyroptosis *in vivo* and *in vitro* dependent on activation of PI3K/AKT and NLRP3 inflammasome. Furthermore, inhibiting the activity of hypoxia-inducible factor (HIF)-1α can reduce the expression of CD73 after SCI. Interestingly, the expression of HIF-1α in microglia was CD73-dependent, meaning that a positive feedback regulatory loop may be formed between CD73 and HIF-1α, which involved in regulating microglial pyroptosis and inhibiting neuroinflammatory response after SCI (Xu et al., 2021). Another study by the same researching group unmasked that toll-like receptor (TLR4) was activated after SCI, promoting the expression of long non-coding RNA (lncRNA)-F630028O10Rik, and also enhanced microglial pyroptosis by activating the PI3K/AKT axis and NLRP3 inflammasome (Xu et al., 2020). Subsequently, they found in TLR 4 knockout mice that TLR4 upregulated the expression of dead-box helicase 3 X-linked (DDX3X) by activating the JAK2/STAT1 pathway, which further aggravated NLPR3-induced pyroptosis in microglia (Wang et al., 2022). Intriguingly, this academic team believes that either the promotion or inhibition of microglia pyroptosis is through the activation of the PI3K/AKT axis in a similar way. Whether it was due to regulation of different PI3K isoforms remains unknown.

Oxidative stress, as an important pathogenic inducement of secondary SCI, refers to the imbalance between the oxidative damage derived from the ROS and the antioxidant defense system. As novel biomarkers of oxidative stress, advanced oxidation protein products (AOPPs) are formed by the biological reaction of chlorinated oxidants (HOCl/OCl^–^) and proteins, including dityrosine- and cross-linking protein products (Skvarilová et al., 2005; Cristani et al., 2016), and involved in different neuroinflammatory diseases, such as Parkinson’s disease, and amyotrophic disease (Pasquali et al., 2015). AOPPs can enhance ROS generation by activating nicotinamide adenine dinucleotide phosphate (NADPH) oxidase. Liu et al. (2020) discovered that the expression levels of AOPPs in plasma, cerebrospinal fluid and spinal cord were significantly elevated. APPOS induced microglia activation *in vivo* after SCI and regulated BV2 cells to generate intracellular ROS by activating NADPH *in vitro*. Subsequent experiments suggested that AOPPs induced activation of the NLRP3 inflammasome, caspase-1, IL-18, and IL-1β and cleavage of GSDMD in BV2 cells through Nox4/ROS/Mitogen-activated protein kinase (MAPK)/NF-κB signaling pathway, ultimately leading to pyroptosis. Afterward, the investigators applied apocynin, the NADPH oxidase inhibitor, in rat hemi-contusion injury model. Results showed that apocynin significantly reduced the level of AOPPs after SCI, attenuated microglia pyroptosis by inhibiting MAPK/NF-κB axis and meanwhile ameliorated forelimb motor function. Another research conducted by this academic group demonstrated that similar to apocynin, kaempferol pretreatment reduced the ROS production by impeding NADPH oxidase 4 and inhibiting the phosphorylation of p38 MAPK and c-Jun N-terminal kinase (JNK), thereby suppressed the nuclear translocation of NF-κB p65 to induce pro-inflammatory cytokines, which subsequently alleviated NLRP3-mediated microglial pyroptosis (Liu et al., 2021). Upregulation of Nrf2 expression facilitates functional recovery after SCI (Xia et al., 2018). Zhang D. et al. (2022) discovered that Nrf2 overexpression reduced pyroptosis after SCI by facilitating miR–146a transcription. Interestingly, GSDMD was a downstream target of miR–146a. MiR–146a knockdown or GSDMD overexpression counteracted the inhibitory effect of Nrf2 on microglial pyroptosis (Zhang D. et al., 2022).

Celastrol and Polygotin are isolated from plants and possesses various biological properties, including anti-inflammatory and neuroprotection. In SCI model, these natural products inhibited the activation of NLRP3 via NF-κB pathway in microglia and alleviated the inflammatory response by reducing the release of inflammatory factors such as tumor necrosis factor (TNF) -α and IL-1β, thus promoted the restoration of limb motor functionality in rats (Dai et al., 2019; Lv et al., 2019). Another plant extract, paeonol, prevents recovery after SCI by inhibiting the pyroptosis of microglia via TLR4/MyD88/NF-κB (p65) pathway (Zhao et al., 2022b).

The above statements collectively indicate that microglia are important mediators of innate immune responses following SCI and are critical for subsequent inflammatory responses. Although the current researches have not fully documented the mechanism of microglial pyroptosis after SCI, it is undeniable that inhibiting microglial pyroptosis has broad application prospects for the treatment of SCI.

### Neuron

Neuron are the basic unit of structure and function of the CNS and the main carrier of neural activity. Pyroptosis performs a key role in neuronal death after SCI, in which GSDMD overexpression can lead to massive neuronal loss (Xu et al., 2021). The most extensively studied inflammasomes in spinal neurons are NLRP3, NLRP1, and AIM2. Accumulating evidence now points to significant anti-inflammatory biological effect of carbon monoxide (CO) (Onyiah et al., 2013; Nikolic et al., 2014). CO releasing molecule-3 (CORM-3), a classical donor of CO, inhibited inositol-requiring enzyme 1(IRE1) phosphorylation in neurons after SCI, reduced the expression levels of NLRP1 and NLRP3, and restrained the activation of caspase-1 and caspase-11, thereby preventing neuronal death and promoting motor function after SCI by alleviating pyroptosis (Zheng et al., 2019). MCC950, an NLRP3-specific inflammasome inhibitor, is a recently developed selective small molecule, and been reported to inhibit the activation of NLRP3 inflammasome in spinal cord tissue and the expression levels of pro-inflammatory factors such as caspase-1, TNF-a, IL-1β, and IL-18 after SCI, as a result, improved the hindlimb motor strength of mice and histopathological score. *In vitro*, MCC950 can also inhibit LPS or oxygen–glucose deprivation (OGD)-induced NLRP3 inflammasome activation and alleviated neuronal damage (Perera et al., 2018; Jiao et al., 2020). Metformin is a commonly used hypoglycemic drug for the treatment of type 2 diabetes with anti-inflammatory, anti-apoptotic and antioxidant properties, therefore it is also a candidate drug for the treatment of neurological diseases. A recent study confirmed that metformin improved motor function recovery after SCI through phosphorylating AMPK and reducing the release of pro-inflammatory cytokines [IL-1β, IL-6, and tumor necrosis factor alpha (TNF-α)] and NLRP3 activation (Yuan et al., 2022).

Zinc is essential for cell physiological processes and involved in the activities of enzymes and transcription factors (Wang et al., 2014). It is known that zinc may inhibit the catalytic activity of NO synthase, thereby reducing oxidative damage and neuroinflammation (Jurowski et al., 2014). Lithium is used to treat bipolar disorder and exerts a neuroprotective role in neurological diseases. In SCI model, both zinc and lithium chloride (LiCl) treatment resulted in down-regulation of NLRP3 inflammasome, ROS and malondialdehyde in spinal cord, which protected against neuroinflammation and motor deficits (Li D. et al., 2020; Zhao et al., 2022d). Interestingly, the anti-pyroptosis effects of these two ions were both via the Nrf2/heme oxygenase-1(Nrf2/HO-1) pathway. Thus, exogenous CORM-3, inhalable CO, MCC950, Metformin, zinc, and LiCl are potential therapeutic targets for SCI.

AIM2 recognizes cytosolic or foreign dsDNA in neurons (Adamczak et al., 2014). Spinal cord ischemia-reperfusion injury (SCIRI) is a common complication after temporary thoracoabdominal aortic occlusion, resulting in motor function and sensory impairment (Miranda et al., 2018). Recent study suggested that motor dysfunction after reperfusion was closely related to elevation of dsDNA levels in serum and cerebrospinal fluid in SCIRI model (Li et al., 2019). Application of AIM2-targeting siRNA blocked the activation of AIM2, which decreased the expression levels of AIM2, ASC, caspase-1, and IL-1β and inhibited neuronal pyroptosis. However, poly(dA)-oligo(dT), nucleotides composed of a 2:1 mix of poly(dA) and 18-mer oligo(dT) (Masuda et al., 2018), can reverse the above effect and accelerate neuronal pyroptosis.

Guo et al. (2022) proposed that PC12 suffering OGD/R or SCIRI could increase the expression level of lncRNA H19 and aggravate neuronal pyroptosis. MiR-181a-5p, a negative regulator of HMGB1, was inhibited by upstream lncRNA H19. LncRNA H19 silence or knockdown alleviated SCIRI induced neuronal pyroptosis by downregulating HMGB1 (Guo et al., 2022). Exosomes (EXOs) are important messengers for intercellular communication in various biological processes. Proteins, DNA fragments, RNAs, lipids and other substances rich in exosomes play a role in intercellular communication (Xin et al., 2021). A recent study indicated that circ_003564, a non-coding circular RNA contained in bone marrow mesenchymal stem cell (BMSC) -derived EXOs, attenuated neuronal NLPR3-related pyroptosis when BMSC-EMO and neuron were co-cultured (Zhao et al., 2022c). Due to the wide variety of circRNAs, they may regulate pyroptosis through various pathways and multiple targets.

Studies carried out by Zhu et al. (2020) implied that the expression levels of caspase-1 and IL-1β in mice were significantly reduced when treated with Smad3 inhibitor, which alleviated inflammatory neuronal death after SCI. Interestingly, the researchers denied that AIM2 or NLRP1 were involved in the regulation of Samd3, but did not confirm which inflammasomes or gasdermins were crucial. Growing evidence has documented that NLRP1 inflammasome can activated by extracellular K^+^ ions via pannexin-1 channels (de Rivero Vaccari et al., 2009) or other intracellular or extracellular substances, such as intracellular ATP depletion, intracellular ion flux downstream of the purinergic P2 × 4/7 receptors and extracellular Ab (Liao and Mogridge, 2013; Chavarría-Smith and Vance, 2015). During the process of CNS injury, both K^+^ efflux and ATP release are engaged in the formation of inflammasomes. Purinergic P2 × 4 receptors are abundantly expressed in neurons. SCI causes excess ATP release and triggers these purinergic receptors, ultimately promoting the formation of the NLRP1 inflammasome (Wang et al., 2004; Zhou et al., 2016). These statements collectively demonstrate that regulation of K^+^ and ATP emerged as a potential way for the treatment of pyroptosis after SCI.

### Crosstalk between microglia and neuron

Under physiological conditions, microglia play the roles of immune surveillance, nourishing neurons and maintaining the homeostasis of the CNS. Nevertheless, the hyperactivation of microglia leads to the production of a large number of pro-inflammatory factors, including nitric oxide (NO), free radicals and ROS, which cause neuronal death (Block et al., 2007). That is, neuron and microglia reciprocally influence each other.

Recent studies identified that mitochondrial ROS (mtROS) and mitochondrial DNA (mtDNA) released from damaged mitochondria in microglia after SCI acted as assembly activators to promote and amplify the activation of NLRP3 (Elliott and Sutterwala, 2015). Quercetin, a natural bioflavonoid, promoted impaired mitochondrial clearance by enhancing microglial mitophagy to reduce mtROS accumulation, NF-kB activation and subsequent NLRP3 inflammasome assembly, thereby reducing neuronal degeneration caused by excessive inflammatory toxicity and exerting neuroprotective effect (Han et al., 2021). The microglial voltage-gated proton channel (Hv1) maintained the intracellular physiological pH and was involved in ROS-induced neuronal damage in ischemic stroke (Tian et al., 2016). Knockdown of Hv1 in microglia reduced ROS production following SCI, then attenuated neuronal apoptosis and NLRP3-inflammasome-mediated pyroptosis, thereby promoting axonal regeneration and improving motor function. In the PC12 cell oxygen-glucose deprivation/reoxygenation (OGD/R) model, NLRP3, ASC, and caspase-1 p20 were significantly up-regulated, contrarily, ROS scavengers could reverse neuronal NLRP3 inflammasome activation and pyroptosis (Li X. et al. 2020). LPS induces BV2 cells to produce IL-6, TNF-α, NO, and ROS and leads to the death of HT22 hippocampal neurons. Ginsenoside Re (Ginsenoside Re) protects hippocampal neurons by inhibiting the production and release of pro-inflammatory mediators in microglia. In addition, LPS induced BV2 cells to produce IL-6, TNF-α, NO, and ROS and led to the death of HT22 hippocampal neurons. Ginsenoside Re prevented hippocampal neurons death by causing reduced microglial activation and pro-inflammatory mediators release in microglia (Madhi et al., 2021). The mechanism of interaction between microglia and neurons following SCI warrants deeper examination, which can provide a more comprehensive theoretical basis for the treatment of SCI.

### Oligodendrocyte

Oligodendrocytes (ODCs) play an important role in the process of myelination and regeneration in the spinal cord to maintain neuronal electrophysiological signal conduction. ODCs are easily affected by the local environment after SCI and undergo necrosis and apoptosis, leading to demyelination and impaired axonal function. Recent studies have revealed that ODCs can undergo caspase-1-dependent pyroptosis. In the multiple sclerosis model, ODCs pyroptosis caused inflammatory demyelinating lesions, however, the caspase-1 small molecule inhibitor VX-765 reversed ODCs pyroptosis and alleviated demyelinating lesions (McKenzie et al., 2018). ODCs and microglial pyroptosis was observed *in vitro* following SCI and was alleviated by estradiol (Zendedel et al., 2018). Differentiation of oligodendrocyte precursor cells (OPCs) into mature oligodendrocytes is a critical step in remyelination. Thioredoxin interacting protein (TXNIP), an endoplasmic reticulum (ER)-induced protein, is increased in SCI. TXNIP is an important linking factor between ER stress and inflammasome, which can trigger the activation of NLRP3 and caspase-1, ultimately activate pyroptosis (Oslowski et al., 2012). ER activates caspase-2 via NLRP3, resulting in mitochondrial dysfunction. Damaged mitochondria release mitochondria-derived DAMPs that activate inflammasomes, leading to caspase-1 activation, and pyroptosis (Zhou et al., 2011; Bronner et al., 2015). Yanagisawa et al. (2019) reported that the expressions of TXNIP, NLRP3, ASC, and caspase-2 were significantly increased in OPCs, while the expression levels were lower in astrocytes. OPCs are less tolerant to ER stress than other cell types. Inhibition of TXNIP and NLRP3 inflammasome provides a crucial means to alleviate the pyroptosis and demyelinating injury of OPCs. Although OPCs pyroptosis can be induced by ER, more studies are needed to confirm whether there are other pathways causing pyroptosis.

### Astrocyte

Astrocytes, the most numerous and widely distributed glial cells in the CNS (Han et al., 2020), play a more active role in maintaining the BBB/BSCB, antioxidant and neural circuit formation (Lin et al., 2020). Current evidence has noted the important role of pyroptosis in astrocytes. Unconjugated bilirubin induced brain astrocyte pyroptosis through a caspase-1-dependent pathway (Feng et al., 2018). Dynamin-related protein 1 (Drp1) mediated mitochondrial fission and triggers NLRP3 inflammasome activation in astrocytes and microglia. FK866 inhibited Drp1-mediated activation of NLRP3 and pyroptosis and protected against cerebral ischemia-reperfusion injury (Zou et al., 2020).

Ethanol induced mitochondrial ROS production and NLRP3/caspase-1-dependent pyroptosis in astrocytes, and this biological effect can be reversed by the ROS scavenger mito-TEMPO, NLRP3 blocking peptide, or the caspase-1 inhibitor Z-YVAD-FMK (Alfonso-Loeches et al., 2014). In the primary neuron-astrocyte co-culture OGD/R model, Zhang et al. (2020) demonstrated that NLRP6 was mainly expressed in astrocytes, with little expression in neurons and microglia. NLRP6 induced astrocytes pyroptosis by activating caspase-1, which aggravated neuronal damage, which NLRP6 silence reversed this effect. One group demonstrated that inflammasome levels in astrocytes were significantly elevated on day 3 after SCI (Yanagisawa et al., 2019). However, astrocytes were resistant to inflammasome-mediated pyroptosis, which may be one of the reasons for the survival of astrocytes after SCI and resulted in the formation of glial scarring (Leal-Filho, 2011).

### Pericyte

Pericytes participate in the formation of blood vessels and maintain the function of endothelial cells. Pericytes are also a kind of pluripotent stem cells, which are engaged in the formation of the blood-brain barrier (BBB) and the blood-spinal cord barrier (BSCB) in the central system. In human brain pericytes, there is no typical inflammasome activation of NLRP1, NLRP2, NLRP3, or NLRC4, but only atypical activation of the nucleotide-binding oligomerization domain containing (Nod) 1, Nod2, and TLR2 (Nyúl-Tóth et al., 2017). SCI inevitably leads to pericyte damage, disruption of microvascular stability, and increased BSCB leakage (Figley et al., 2014). The therapeutic potential of stem cell-derived exosomes has attracted increasing attention as they cannot only exert stem cell-like paracrine functions but also overcome the limitations of stem cell transplantation (Wu D. et al., 2021). BMSC-derived EXOs significantly reduced the expression of caspase-1 and IL-1β in pericytes after SCI, and increased the pericyte/endothelial cell coverage of the vessel wall and BSCB integrity, ultimately, prevented myelin loss and spinal neuron death. BMSC-EXOs were confirmed to improve BSCB integrity by inhibiting the activation of Nod1 inflammasome and pericyte pyroptosis (Zhou Y. et al., 2022). Inhibition of pericyte pyroptosis to alleviate BSCB leakage after SCI warrants deeper examination.

## Interaction between regulated cell death in spinal cord injury

RCD is an important way of CNS development, maintaining homeostasis and removing damaged cells. Dozens of types of RCD exist, ranging from non-inflammatory (e.g., autophagy, apoptosis) to proinflammatory (e.g., pyroptosis, ferroptosis, necroptosis), which is fundamentally different from necrosis (Niu et al., 2022). It bears consideration that each RCD is defined by a distinct molecular signature and may interact with each other. Single stimulus factor may trigger multiple forms of cell death programs in a given cell population.

### Interaction between pyroptosis and autophagy

Autophagy is an evolutionarily conserved catabolic process that degrades damaged proteins and organelles through lysosomes. The role of autophagy in CNS injury is considered a double-edged sword. On the one hand, Autophagy maintains cellular homeostasis by recycling energy and substances, on the other hand, excessive or inappropriate autophagy may induce autophagic cell death (Chen et al., 2012). There are various crosstalks between pyroptosis and autophagy, especially with the mitochondria as the core organelle. Several groups have visualized that autophagy can negatively regulate NLRP3 inflammasome activation by removing endogenous activators of the inflammasome, including ROS from damaged mitochondria, inflammatory factors or cytokines. Meanwhile, the NLRP3 inflammasome can restrain autophagy induced by TLR4-Toll/IL-1R domain-containing adaptor-inducing IFN-β (TRIF) signaling pathway by activating caspase-1 to cleave the TIR domain adaptor protein (Jabir et al., 2014; Lai et al., 2018). Studies have shown that cathepsin B (CTSB), a cathepsin released from lysosomal rupture, not only directly activates NLRP3 (Chevriaux et al., 2020), but also inhibits autophagy by regulating the activity of the kinase ULK1, which negatively regulates the activation of the NLRP3 (Qi et al., 2016; Zhao et al., 2021). Recent studies have implicated that inhibition of the NLRP3 inflammasome by enhancing autophagy prevented neuronal deficits during cerebral ischemia-reperfusion (He et al., 2017; Wang et al., 2019). In addition, inflammasomes can also inhibit mitophagy in macrophages in a caspase-1-dependent manner, leading to mitochondrial dysfunction (Zhao et al., 2021). While most investigators believed that autophagy reduced inflammatory injury by inhibiting the inflammasome, some scholars have found that autophagy can promote the activation of the NLRP3 in yeast cells (Dupont et al., 2011). Accordingly, a complex interaction mechanism may exist between pyroptosis and autophagy.

Accumulating evidence suggests that secondary injury after SCI is closely related to mitochondrial damage and excessive ROS production (Chen X. et al., 2018). In a SCI model of mice, treatment with piperine, a natural compound present in spices, has been found to prevent neurons from SCI by inhibiting oxidative stress, and pyroptosis, mediated by the promotion of autophagy (Zhang H. et al., 2022). Damaged mitochondria lead to massive accumulation of ROS, which induces NLRP3 inflammasome formation and subsequent activation of caspase-1-dependent pyroptosis (Lin et al., 2019). In addition, mtDNA released into the cytoplasm is also an important activator of the inflammasomes. ROS oxidation leads to the opening of the mitochondrial permeability transition (MPT) pore, through which mtDNA is released into the cytoplasm (Nakahira et al., 2011; Yu et al., 2019a). Although one research has demonstrated that the oxidized form of mtDNA mainly activates NLRP3, and the non-oxidized form of mtDNA mainly tends to activate the AIM2 inflammasome, these have not been further confirmed in subsequent studies (Shimada et al., 2012). Mitophagy, a selective autophagic degradation of damaged mitochondria, can reduce the accumulation of ROS and the release of mitochondria-related damage factors (Li et al., 2018; Chu, 2019), just like the protective mechanism of quercetin mentioned above. In support of this hypothesis, it was recently elucidated that betulinic acid restored autophagy flux in neuron following SCI, which accelerated mitophagy to eliminate ROS accumulation and inhibit pyroptosis. As an important member of the MiT family, the transcription factor EB (TFEB) is involved in the regulation of the lysosome-autophagosome pathway. Betulinic acid induced phosphorylation of 5′ adenosine-monophosphate-activated protein kinase (AMPK) and inhibited mammalian target of rapamycin (mTOR) phosphorylation. Briefly, betulinic acid enhanced neuronal mitophagy and inhibited pyroptosis through the AMPK-mTOR-TFEB signaling pathway to promote functional recovery following SCI (Wu C. et al., 2021). Another research by the same study group confirmed that baicalein, namely 5,6,7-trihydroxyflavone, a component derived from the root of the herb scutellaria baicalensis, inhibited NLRP3 inflammasome activation and ER stress-mediated apoptosis via promoting neuronal autophagy to attenuate spinal cord defect-reperfusion injury. This neuroprotective effect could be counteracted by the autophagy-specific inhibitor 3MA (Wu et al., 2020). Although autophagy plays a vital role in neuroinflammation by regulating the inflammasome, some questions remain to be elucidated, such as whether autophagy can affect pyroptosis through other pathways or inhibiting neuroinflammation by promoting autophagy brings additional damage? With the research continued, it may provide new methods for the treatment of SCI by regulating pyroptosis and autophagy.

### Interaction between pyroptosis and apoptosis

Apoptosis is one of the most common forms of RCD in SCI, in which cell membrane integrity is maintained, whereas pyroptosis involves rapid rupture of membrane integrity. Molecular interactions between pyroptotic and apoptosis signaling pathways are becoming increasingly apparent. Numerous markers of apoptosis are shared with pyroptosis owing to the overlaps, including TUNEL (Jorgensen and Miao, 2015), Annexin-V positivity (de Vasconcelos et al., 2019), and PARP cleavage (Taabazuing et al., 2017). Annexin-V-positive and PI-negative dual-labeling paradigm are considered to characterize early apoptotic cells, but similar labels can also be identified in pyroptotic cells after either NLRP1 or NLRC4 activation (de Vasconcelos et al., 2019).

Early studies have shown that activation of executioner caspases, principally caspase-3, have long been considered important markers of apoptosis (Mattson, 2000). Apoptotic stimuli can trigger caspase-3-mediated inactivation of GSDMD by generating a non-pore-forming truncated N-terminus, thereby preventing pyroptosis. Intriguingly, the cleavage of GSDMD by caspase-3 is an inactivating event (Taabazuing et al., 2017). However, It has also been shown that activated caspase-3 can trigger the cleavage of GSDME and induce pyroptosis (Rogers et al., 2017). Caspase-8 is also an important participator in the cell death cascade. Caspase-8 can cleave and activate both GSDMD and GSDME to cause pyroptosis (Sarhan et al., 2018; Chen et al., 2019). Activated caspase-8 can drive classical caspase-dependent apoptosis and inhibit recombinant receptor-interacting serine-threonine kinase 3 (RIPK3)-mediated necroptosis. In addition, caspase-8 can promote NLRP3 activation by cleaving GSDMD to induce pyroptosis, a process that does not require RIPK3 kinase activity (Bertheloot et al., 2021). Furthermore, caspase-8 also plays a key role in pro-IL-1β synthesis and maturation (Moriwaki et al., 2016).

By contrast, caspase-1 is the important checkpoint in the regulation of apoptosis by pyroptosis. One academic team has elucidated caspase-1-dependent mitochondrial depolarization and cytochrome C release downstream of NLRP3 and AIM2 inflammasomes (Yu et al., 2014). There is some evidence that apoptotic executioner caspase-3 and caspase-7, the caspase-1 substrates, are proteolytically active during pyroptosis (Taabazuing et al., 2017). Caspase-1 can sensitize caspases-3/7, either directly through proteolysis or indirectly through activation of the upstream activator caspases-8/9 (McKenzie et al., 2020). Nevertheless, in the absence of the proteins required for pyroptosis can prompt cells toward apoptosis rather than pyroptosis. Pyroptotic stimuli can induct caspase-1-dependent apoptosis in the absence of GSDMD (DiPeso et al., 2017; Taabazuing et al., 2017; Tsuchiya et al., 2019) or caspase-1-independent apoptosis in the absence of caspase-1 (Sagulenko et al., 2013). Nonetheless, evidence for activation of caspase-3/7 downstream of caspase-1 in GSDMD-mediated pyroptosis is abundant. The unifying mechanism underlying the interactions between pyroptotic and apoptotic signaling pathways warrant further investigation in the context of SCI.

## Conclusion

SCI is one of the most serious traumas that not only reduces the quality of life and the activity level of patients, but also imposes a heavy financial burden on families and society. Although years of research have yielded some fruitful approaches, great challenges remain in the treatment of this complex and multifaceted disease. Neuroinflammation is the central process of secondary phase of SCI after initial cell death. Pyroptosis induced by intense inflammatory response accelerates the process of cell death and inhibits the recovery of nerve function after injury (Okada, 2016; Chen L. et al., 2018). Researchers have explored a variety of methods and drugs to inhibit pyroptosis after SCI to alleviate neurological dysfunction, opening a new channel for clinical treatment of SCI, but the molecular mechanism of pyroptosis remains to be further investigated.

Nevertheless, less research has been done on other inflammasome receptors during SCI, such as Pyrin (detects changes in cellular homeostasis mediated by bacterial toxins) (de Torre-Minguela et al., 2017), NLRP2 (responds to extracellular ATP) (Minkiewicz et al., 2013), NLRP6 (Has a negative regulatory effect on CNS) (Anand et al., 2012), NLRC5 (responds to bacterial ligands and DAMPs) (Davis et al., 2011). In addition, NLRX1, a member of NLR-family proteins, have immunomodulatory effects independent of inflammasome formation (Guo et al., 2016; Gharagozloo et al., 2019). Pyroptosis typically involves the cleavage and activation of GSDMD by caspase-1, with notable exceptions including caspase-8/3 (Wang et al., 2017; Orning et al., 2018; Sarhan et al., 2018). Although there is evidence that caspase-8 activation can induce NLRP3 inflammasome-triggered pyroptosis, and the caspase-8/GSDMD signaling pathway may be another initiating factor of pyroptosis (Vince and Silke, 2016), the mechanism of action of casapase-8/3-dependent pyroptosis in SCI remains unclear. At present, the research on pyroptosis after SCI mainly focuses on the caspase-1-dependent pathway, and the caspase-4/5/11-dependent pathway needs additional research. Similar to the regulation of pyroptosis by GSDMD, secondary necrosis mediated by GSDME (Aizawa et al., 2020) or GSDMA (Zhao et al., 2022a), as well as GSDMD mediated by GzmA can also induce pyroptosis, but there are few related experimental studies concerning SCI. Whether these pyroptosis signaling pathway are active in SCI remains to be determined.

Multiple cell death mechanisms can affect concurrently on the progression of SCI. A systematic comparison of different cell death modalities in SCI is also something to be expanded in future work. Recent evidence has implicated that NLRP3 inflammasome activation not only contributes to pyroptosis, but also contributes to other types of cell death, such as apoptosis, necroptosis, and ferroptosis (Huang et al., 2021). Notably, not all investigations point to similar conclusions regarding inflammasome activation and pyroptosis in different cell types. Most studies focus on only one type of cell exposure to inflammation-relevant stimuli, the mechanism of the crosstalk between neuron and glial cells within SCI is still unclear. Species, strains, ages, animal model, *ex vivo* isolation methods as well as culture conditions likely impact the experimental results. A brief overview of strategies for the inhibition of pyroptosis, such as ROS scavenger (e.g., prussian blue nanozyme) (Ma et al., 2022), caspase-1 inhibitors (e.g., VX-765, VX-740, and Z-YVAD-FMK) (Linton, 2005; Cornelis et al., 2007; Zhang et al., 2019), NLRP3 inhibitors (e.g., MCC950, CY09, OLT1177, Bay 11-7082, Tranilast, Rubesin, and microRNAs) (Juliana et al., 2010; Coll et al., 2015; Huang et al., 2018; Marchetti et al., 2018; Yang et al., 2019; Yu et al., 2019b; Cheng et al., 2021) and GSDMD inhibitors (e.g., disulfiram, Ac-FLTD-CMK) (Yang et al., 2018; Tan C. et al., 2022) are summarized. Additionally, NIMA-related kinase 7 (NEK7), a mitotic serine/threonine kinase and an upstream regulator of NLRP3 inflammasome activation, tend to be a point of intervention (Zhou C. et al., 2022). These compounds tend to have acceptable safety profiles *in vivo* and targeting pyroptosis has already established beneficial outcomes in preclinical models. As touched upon earlier, as a biomarker of SCI neuropathogenesis, the role of NINJ in CNS inflammation is still controversial. Given the important role of NINJ1 in PMR and pyroptosis, reducing neuroinflammation by modulating NINJ may also become a potentially effective approach. Strikingly, knockout or silencing of GSDMs is an important method to attenuate pyroptosis, which has been widely used in the treatment of tumors and pulmonary inflammation (Song et al., 2022; Tan G. et al., 2022), nevertheless, it is rarely used in SCI research and deserves further exploration. In addition, lncRNAs and EXOs are promising regulators of inflammation and pyroptosis during SCI. This work highlights key developments in pyroptosis as a driver of neuroinflammation during SCI and its potential therapeutic approaches targeting pyroptosis. A comprehensive understanding of pyroptosis within SCI may help develop viable neuroprotective strategies.

## Author contributions

JY and GG: writing the manuscript. WW: modification of the manuscript. XL: guidance and revision of the manuscript. All authors contributed to the article and approved the submitted version.
